# 

**Published:** 1855-02

**Authors:** 


"*Reese's* Medical Gazette" has changed its name, and now hails as the "*American* Medical Gazette." This change is made with reference to its "national circulation." So we have now in Gotham, not only a medical college, but a medical journal, both of which are "national institutions." H.

